# Use of Laser in Sleep Disorders: A Review on Low Laser Uvulopalatoplasty

**DOI:** 10.1155/2021/8821073

**Published:** 2021-02-28

**Authors:** Mayank Kakkar, Shaima Malik, Bhumija Gupta, Nikhilesh Vaid, Robby George, Shilpa Singh

**Affiliations:** ^1^Department of General Dentistry, Eastman Institute for Oral Health, University of Rochester, New York, USA; ^2^Department of Orthodontics and Dentofacial Orthopedics, Eastman Institute for Oral Health, Rochester, New York, USA; ^3^Private Dentist, Rochester, , New York, USA; ^4^Department of Orthodontics, European University College, Dubai, UAE; ^5^Department of Orofacial Pain/TMD and Community Dentistry, Eastman Institute for Oral Health, Rochester, New York, USA

## Abstract

**Methods:**

A comprehensive and systematic literature review was conducted using PubMed, Google Scholar, Cochrane Central Register of Controlled Trials, Embase, Web of Science, the US National Institutes of Health Trials Registry, WHO Library, and Medline. The search strategies were developed to cover publications from January 2010 through March 2020. The past 10 years of the search were performed to report the data following systematic review and meta-analysis protocol (PRISMA-P) 2015 statement.

**Results:**

With the help of keywords, the total number of abstracts identified was 946. These abstracts were further reviewed as per inclusion and exclusion criteria, and 106 abstracts were identified to match the selection criteria. Further review of full articles resulted in 12 articles that matched the inclusion criteria for the study.

**Conclusion:**

Er:YAG can be a good alternative and least invasive therapy for managing snoring and obstructive sleep apnea. Er:YAG therapy is considered to nonsurgical intervention with minimum side effects and can be performed chairside.

## 1. Introduction

Sleep-disordered breathing (SDB) comprises a range of disorders with varying degrees of significance and severity including habitual snoring, upper airway resistance syndrome (UARS), and obstructive sleep apnea (OSA) [1]. Obstructive sleep apnea (OSA) is a sleep disorder in which breathing is compromised briefly during sleep [2, 3]. There is resistance of airflow during sleep due to partial or complete collapse of the upper airway mainly the oropharyngeal tract. It occurs due to failure of the muscle to keep the airway open or that supports the soft tissue in the throat such as tongue and soft palate [2, 4]. It is estimated that 4% of men and 2% of women meet the diagnosis criteria for obstructive sleep apnea in the United States with prevalence reported to be 14% of men and 5% of women. Prevalence is found to be higher in the Hispanic, Asian, and African American populations [5]. Snoring is a part of sleep-disordered breathing which may be a symptom of obstructive sleep apnea; however, not all patients who snore may have clinically evident obstructive sleep apnea [6]. Obesity, male gender, advancing age, and mandibular-maxillary insufficiency are well-characterized risk factors. Risk factors include snoring, male gender, middle age, menopause in women, obesity, and a variety of craniofacial and oropharyngeal features such as a large neck circumference, retro- or micrognazia, nasal obstruction, enlarged tonsils/adenoids, macroglossia, and low-lying soft palate (Guilleminault and Quo, 2001; Dempsey et al. 2010). Due to monotonous upper airway constriction during sleep, it may result in abnormally slow or shallowing breathing (hypopnea) and temporary cessation of breathing (apnea) which will deprive the adequate oxygen supply (hypoxia), excessive carbon dioxide (hypercapnia), and sleep fragmentation [1]. Consequently, this leads to OSA syndrome with subjective symptoms of daytime sleepiness, cognitive and neurobehavioral dysfunction, inability to concentrate, memory impairment, and mood changes such as irritability and depression [1, 7, 8], eventually affecting the quality of life. The most common symptoms during sleep are snoring, witnessed apneas, choking at night, nocturia, and insomnia. Chronicity of sleep apnea affects the function of different organs and systems most importantly the brain and cardiovascular system thereby altering body metabolic balance (Bradley et al. 2008). Uncontrolled medication-resistant hypertension may be one of the risks of sleep apneas too (Bradley et al. 2008). Other cardiovascular disorders may include ischemic heart diseases, cardiac arrhythmias, and stroke (Bradley et al. 2008). Sleep-related hypoxia is also known to induce low-grade systemic inflammation (Jordan et al. 2014), results in hyperalgesia effect, and enhances pain sensitivity [9]. Chronic pain conditions are few other comorbidities like fibromyalgia, temporomandibular disorders, and headaches [9–12]. The most common symptoms associated with OSA are illustrated in Table 1.

## 2. Material and Methods

This review and search were performed with the intention to report the data in accordance with systematic review and meta-analysis protocol (PRISMA-P) 2015 statement.

### 2.1. Search Strategy

A comprehensive initial systematic literature review was conducted using the PubMed databases and Google Scholar. This systematic search includes PubMed and Medline (2010-present), Cochrane Central Register of Controlled Trials, Embase, Web of Science, Google Scholar, the US National Institutes of Health Trials Registry, WHO Library, and Medline with no language filter. Additional dental organization websites were searched, including the American Dental Association, to identify articles and statistics that evaluated the effectiveness of the low laser on uvulopalatoplasty/soft palate in sleep apnea patients and compared the effectiveness of Er:YAG and Nd:YAG lasers in uvulopalatoplasty. The search strategies were developed to cover publications from January 2010 through March 2020. The past 10 years of search were selected as per investigator's decision and the use of laser-assisted uvulopalatoplasty as treatment for apnea/snoring.

### 2.2. Selection Criteria

The primary search objective was to identify all papers reporting the results of (1) randomized clinical trials (RCT) of laser-assisted uvulopalatoplasty in the treatment of adults with OSA or snoring or sleep apnea and (2) both randomized/nonrandomized clinical trials and case reports on the laser-assisted uvulopalatoplasty in OSA/sleep apnea adults. The first step was to identify and review all of the studies listed for analysis in three major literature reviews, a Cochrane Collaboration review [1] and a second systematic literature review published by the National Institutes of Health Research (NIHR) [2] on the use of CPAP for the treatment of OSA, and a second Cochrane Collaboration review on its surgical management [3]. The second step was an extensive search of the PubMed/Medline database, initiated using the following combined search terms: “Laser and obstructive sleep apnea” (*n* = 268); “Laser and snoring” (*n* = 265); and “Er:YAG and snoring” (*n* = 5); laser and sleep apnea (*n* = 380); Nd:YAG and sleep apnea (*n* = 10); Nd:YAG and snoring (*n* = 7); Nd:YAG and obstructive sleep apnea (*n* = 8); Nd:YAG and uvula (*n* = 8). From these lists, studies were identified that (a) did not replicate studies already found and (b) were otherwise eligible for inclusion. The third and final step was a review of all reference lists and tables of other studies found within papers identified in the second step.

Articles were considered for inclusion into the study by reviewing the titles and abstracts of all retrieved studies. The senior study authors SS, BG, and MK did this, and results were compiled to ensure no studies were missed. The full text of selected studies was then analyzed to ensure that the following inclusion criteria were met: diagnosis of obstructive sleep apnea, no confounding data for central sleep apnea, and the paper referred to laser-assisted uvulopalatoplasty in OSA or snoring.

### 2.3. Search Terms/Keywords

Laser, sleep apnea, Nd:YAG, snoring, obstructive sleep apnea, uvula, and uvulopalatoplasty.

### 2.4. Inclusion Criteria

The literature included in this study was based on the following inclusion criteria:

1. Studies/case reports on laser-assisted uvulopalatoplasty as management for snoring or sleep apnea
2. Randomized clinical trials on laser-assisted uvulopalatoplasty for management of sleep apnea or snoring
3. Studies/literatures or case reports for management of sleep apnea by laser in the soft palate

### 2.5. Exclusion Criteria

The literature eligible for inclusion in this study was based on the following exclusion criteria:

1. Literature/studies or case reports on conventional surgical management of sleep apnea
2. Literatures/studies reporting the laser-assisted surgical management strategies in combination including trachea, soft palate, or uvulopalatoplasty
3. Literature on previous history of wisdom teeth extraction

### 2.6. Selection of Studies and Data Extraction

**Figure 1:**
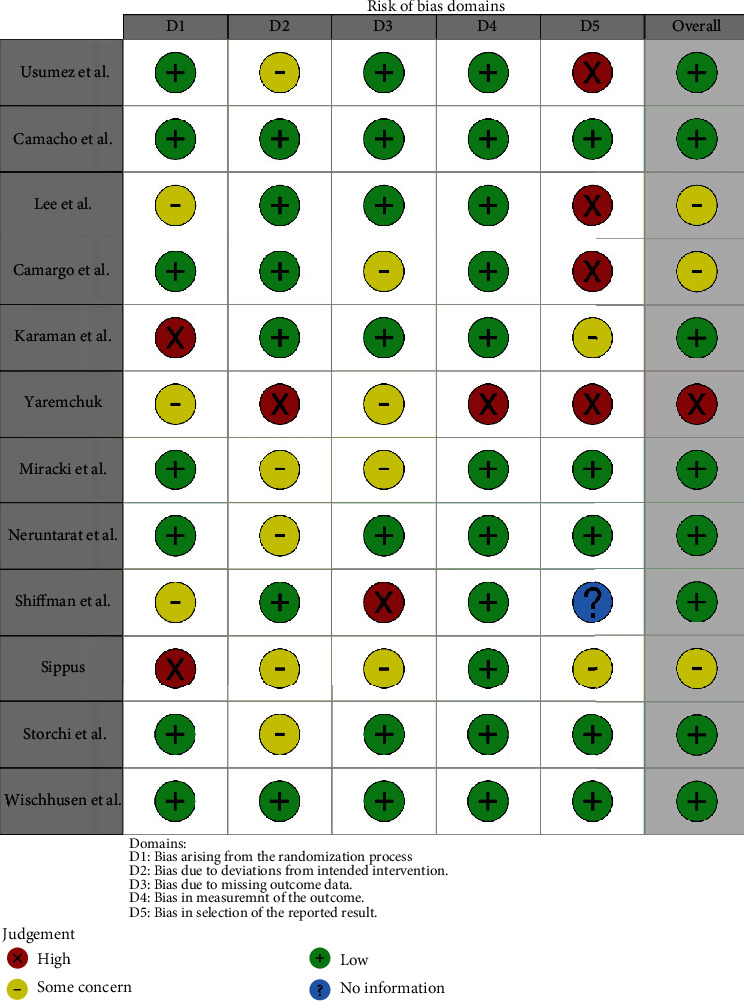
Risk of bias.

The articles were evaluated for their relevance based on the titles and abstracts. Further validation of the articles was done by obtaining the full text of the possible relevant studies that met the inclusion criteria. All the articles were reviewed by three reviewers (SS, BG, and MK). The studies assessed by SS and deemed eligible were checked by MK and BG for methodological quality and inclusion criteria. All disagreements were resolved verbally, with strict adherence to the predetermined inclusion criteria. Risk of bias analysis has been done in Figure 1 [13].

### 2.7. Diagnosis

An appropriate diagnosis of sleep disorders starts with comprehensive examination that includes complete medical history and physical examination. Usually, the diagnosis of sleep disorders happens in either of the three settings: first, usually routine physical examination or dental examination; second, as part of an evaluation of symptoms of snoring or apneas; third, as part of the comprehensive evaluation of patients at high risk for OSA.

Routine examination includes comprehensive sleep history, Epworth sleepiness scale questionnaire, and intraoral, extraoral, and physical examination. Sleep history and ESS will provide details for the sleep quality and subjective symptoms. Physical, intraoral, and extraoral examination will suggest respiratory, cardiovascular, and neurologic symptoms. Narrowing of upper respiratory symptoms can be evaluated by obtaining neck circumference (>17 inches in men and >16 inches in women), body mass index (BMI) ≥ 30 kg/m^2^, a modified Mallampati score of 3 or 4,7 the presence of retrognathia, lateral peritonsillar narrowing, macroglossia, tonsillar hypertrophy, elongated/enlarged uvula, high arched/narrow hard palate, nasal abnormalities (polyps, deviation, valve abnormalities, and turbinate hypertrophy), and/or overjet.

Comprehensive examination can help to identify the risk of OSA. High-risk patients need objective testing to further confirm the severity of the OSA. OSA is diagnosed by performing a sleep study using polysomnography, in which the patient is attached to equipment while sleeping which monitors the oxygen saturation level, oral and nasal airflow, electrocardiographic measurements, and body movements [6]. Based on the apnea-hypopnea index (AHI), and oxygen saturation, sleep apnea can be categorized into 3 groups (refer to Table 2).

### 2.8. Treatment Modalities

OSA should be considered as a chronic condition that requires long-term multidisciplinary management. This includes medical, behavioral, and surgical options to manage OSA. At times, adjunctive therapies can be incorporated to supplement the treatment options. CPAP (continuous positive airway pressure) is considered the gold standard choice of treatment for mild, moderate to severe patients. Alternative therapies are provided based on risk factors and preferences.

Alternative therapies include oral appliances that can be used as adjunctive therapy or in cases of noncompliance to other treatment options. Surgical options include a variety of upper airway reconstructive or bypass procedures, often site-directed and/or staged (LJ Epstein, D Kristo, PJ Strollo et al. 2009) [14]. Evaluation for primary surgical treatment can be considered in patients with mild OSA who have severe obstructing anatomy that is surgically correctible (e.g., tonsillar hypertrophy obstructing the pharyngeal airway) (LJ Epstein, D Kristo, PJ Strollo et al. 2009) [14]. Surgical procedures may be considered as a secondary treatment for OSA when the outcome of PAP therapy is inadequate, such as when the patient is intolerant of PAP, or PAP therapy is unable to eliminate OSA (LJ Epstein, D Kristo, PJ Strollo et al. 2009) [14]. Surgery may also be considered as a secondary therapy when there is an inadequate treatment outcome with an OA, when the patient is intolerant of the OA, or the OA therapy provides unacceptable improvement of clinical outcomes of OSA (LJ Epstein, D Kristo, PJ Strollo et al. 2009) [14]. Surgery may also be considered as an adjunct therapy when obstructive anatomy or functional deficiencies compromise other therapies or to improve tolerance of other OSA treatments (LJ Epstein, D Kristo, PJ Strollo et al. 2009) [9].

Laser-assisted uvulopalatoplasty (LAUP) has been reported as one of the treatment modalities for snoring and OSA. Dr. Kamami in the year 1990 reported the use of a carbon dioxide (CO_2_) laser on 31 patients. She used the CO_2_ laser to erode the soft palate as a treatment for snoring [10]. The LAUP technique eliminates or diminishes the oropharynx obstructions by successfully stripping the colonization of vibrating soft palate, wide posterior tonsil pillars, and excessive posterior mucosa [14]. LAUP was originally carried out with the patient placed in a seated position as clinic procedure under the local anesthesia, with CO_2_ laser vaporization of the “wide lateral pharyngeal walls and low arched soft palate, on both sides of the uvula, sparing the uvula” [14]. In 1994, Dr. Kamami published another research paper with emphasis on the indication of LAUP for OSA patients with 40 of 46 patients being classified as responders [11].

In 2000, the American Academy of Sleep Medicine's Standards of Practice Committee published updated parameters for the use of LAUP (AASM's Practice Parameters for LAUP) and stated that “LAUP is not recommended for the treatment of sleep-disordered breathing” [12]. In this review, we aim to find the effectiveness of the low laser on uvulopalatoplasty/soft palate in sleep apnea patients and snoring. Also, this study aims to touch base on the effectiveness of the Er:YAG and combined use of Er:YAG and Nd:YAG lasers for the uvulopalatoplasty.

## 3. Results

**Figure 2:**
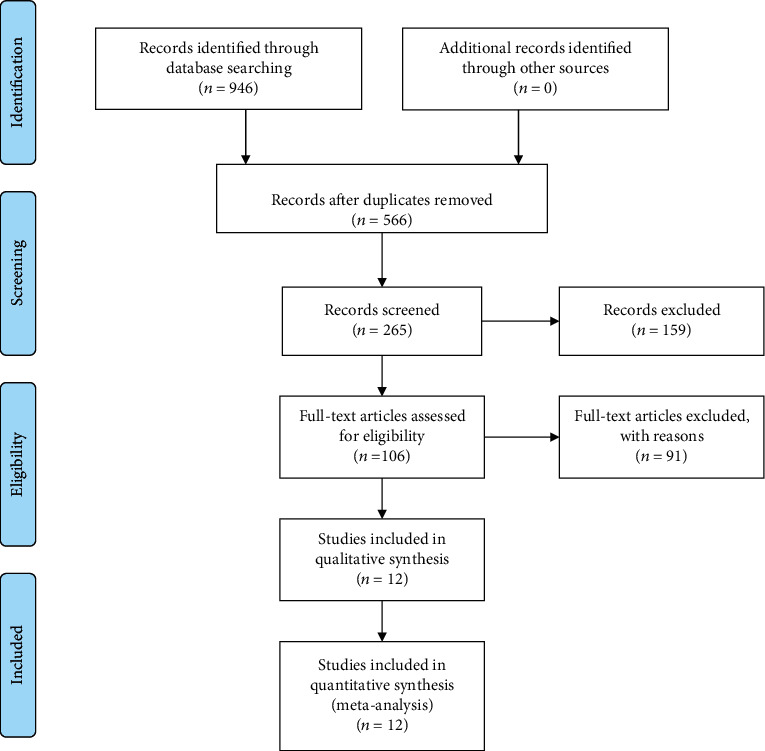
PRISMA 2009 flow diagram.

With the help of keywords, the total number of abstracts identified was 946. These abstracts were further reviewed as per inclusion and exclusion criteria, and 106 abstracts were identified to match the selection criteria. Further review of full articles resulted in 12 articles that matched our inclusion criteria. 91 articles were excluded. There were no articles identified in our manual search. The flow chart in Figure 2 depicts the search strategy for this study. All the included studies and demographics are depicted in Table 3.

## 4. Discussion

Camargo et al. have ongoing research in the recruitment stage. This research has proposed for low laser therapy (LLT/biomodulation) as a therapeutic option for the treatment of snoring and OSA. According to Camargo et al., it can be proved to be efficient, cost effective, and adjuvant therapy to conventional treatments with CPAP. In this study, 250 Mw (mill watts) of laser will be used and a continuous wavelength of 808 nm will be administered on the soft palate, palatine tonsils, pharyngeal walls, uvula, and the base of the tongue. This study hypothesizes that when LLT is applied to the soft palate, it can decrease the phallus collapsibility and snoring and thereby improves the AHI (apnea/hypopnea) index [15].

A prospective study was conducted on 40 patients by Storchi et al. in 2018, where patients with snoring and sleep disorders were evaluated for the effectiveness of Er:YAG (2940 nm) laser treatment sessions. After the three laser sessions, 85% of the patients self-reported to be satisfied after the treatment [16, 17]. The results demonstrated that change in patient's quality of sleep is significantly increased (*p* < 0.0001), snoring severity is significantly improved (*p* < 0.0001), and immediate sensation of breathing improvement (75%) is reported in pre- and postlaser treatment [10]. In addition to this, when applied on the soft palate, uvula, palatine tonsils, and the base of the tongue, the rate of apnea and hypoxia is improved. The mechanism of action of the erbium:YAG laser is a photothermal effect that creates depletion of the collagen fibers in the treated oral mucosa and stimulates a neocollagenase via heat shock protein (HSP) action [9].

In the pilot study conducted by Lee et al. (2015), seven patients were recruited for the study, out of which 5 were diagnosed with OSA and 2 were documented as snorers. Each patient was laser-treated on the palatoglossal arch, palatopharyngeal arch, and uvula using the Er:YAG laser (LightWalker laser, Technology4Medicine, Irvine, CA) having an infrared of wavelength 2940 nm. Prelaser and postlaser CBCT scans were compared, and it was found that laser procedure increased the mean total airway volume from 10.23 ± 0.94 mL to 12.54 ± 1.01 mL (*p* = 0.0179), and the minimum cross-sectional area from 109.7 ± 20.6 mm^2^ to 142.4 ± 29.2 mm^2^ (*p* = 0.0484). The study concluded that low-level laser therapy significantly increases the oropharyngeal airway volume which may serve as an alternative or adjunctive treatment option for sleep-disordered breathing especially CPAP intolerant individuals [18, 19].

Sleep disorder breathing is a broader term which includes OSA and snoring, and there are different studies that have included either one of the diagnoses or both for the study purpose (refer to Table 4).

CPAP remains a treatment option for sleep apnea; however, many patients have reported difficulty adapting to the CPAP machine. CPAP therapy is expensive, and noncompliance rate is higher. Nasal or throat dryness, noise of machine, and size of machine are few of the reasons for which patients tend not to be regular with CPAP use. Due to the physical impairment, CPAP machine may not be an effective option and thus low-level laser therapy (LLLT) can play an important adjunct treatment modality for these populations. Additionally, LLLT is a safe, easy to perform, and inexpensive treatment when compared to CPAP therapy. There has been no serious complication reported to this therapy. LLLT is a simple, minimally invasive, reliable, and painless procedure that can be performed without hospital admission. Application of LLLT use in the soft palate region can act as adjunctive therapy to CPAP. Most of the studies have used a LightWalker AT laser manufactured by Fontana which has a dual-wavelength capacity and can operate with Er:YAG (wavelength 2940 nm) and Nd:YAG (wavelength 1064 nm). Most of the studies have suggested that there are no to minimal complications; however, a systematic review conducted by Wischhusen et al. in 2019 has suggested that there may be some transient complications such as bleeding, dryness, candidiasis, dehiscence, and surgical site infections. The major limitation of this study is that it did not mention about the kind of laser that was used and thus it is hard to comment that these complications are associated with which type of laser or the wavelength. Also, the location where LLLT is applied also is another factor for post complications. The study also suggests that collecting and gathering information such as timing, duration of the surgery, surgical technique, and extend of the surgery can better assess risk and cautions can take while doing the procedures [20].

The study conducted by Shiffman et al. in 2018 aimed at providing the clinical experience with the combined Er:YAG and Nd:YAG laser for the LAUP procedure in the SDB (sleeping disorder breathing) patients. In this study, they have described the LAUP procedure with first using the Nd:YAG laser which acts as an effective coagulator and after that, they have used the Er YAG laser on the same site to further enhance the shrinkage of collagen and neocollagenase resulting in tightening of the soft palate and surrounding tissues. This combined approach of two lasers has been described as NightLase® LAUP protocol [21]. Type of lasers, target location, and wavelength used in the studies are included in Table 5.

Üsümez et al. 2016 performed LLLT Er:YAG on the soft palate of Wistar albino rats with successful contraction of the soft palate. The finding of this study suggests that there is contraction of soft palate tissue but relapse is noticed in the 5^th^ week. This study concluded that Er:YAG application on the soft palate for snoring can be considered to be a safe treatment due to the absence of any carbonization, necrosis, or hemorrhage. Similarly, Dovsak et al. conducted a pilot study and showed that Er:YAG laser treatment is a safe method and is easily tolerated by patients [11].

Sippus et al. 2015 study used Er:YAG laser with HP PS04 handpiece with minimal invasive settings. In these case reports, the laser beam of 10 Hz in LP mode was fired on the soft tissue horizontally or vertically repeatedly in short intervals with obvious shrinkage of soft tissue in the palatal region. Total delivered pulses vary per patient from between 10,000 and 15,000. According to this study, Er:YAG application is a safe and successful treatment in reducing snoring. Its easy application and no anesthesia use make this treatment very well accepted by the patients as well.

## 5. Conclusion

Based on the finding of this systematic review, it is recommended that low-level laser therapy (LLLT) such as Er:YAG lasers can be performed as a treatment modality for sleep-disordered breathing, especially obstructive sleep apnea and snoring. Laser-assisted uvulopalatoplasty (LAUP) can be performed on the pharyngeal and palatal soft tissues to diminish or eliminate the obstructions. This nonsurgical therapy is revolutionary development in treating obstructive sleep apnea patients, especially those who have difficulty in using the CPAP machine. The long-term efficacy, quality of life, and painless laser procedure can be performed with successful outcomes on sleep apnea patients. Based on the research, there have not been any serious complications that were reported that make this procedure an effective treatment modality for sleep apnea patients. Another important outcome of this research is that there needs to a patient selection and inclusion criteria for the LAUP procedure as it may not be suitable for all the patients. It is recommended that there should be more randomized clinical controlled trials in the future with long-term follow-up to check for the LAUP when used in conjunct and adjunct to CPAP machine.

Based on current evidence, Er:YAG can be a good alternative and least invasive therapy for managing snoring. This therapy can also be beneficial as nonsurgical management for sleep-disordered breathing and more importantly in noncompliant CPAP individuals. Er:YAG therapy is considered to nonsurgical intervention with minimum side effects and can be performed chairside. This treatment involved no chemical/pharmacotherapy and no anesthesia or at the most topical or local anesthesia. The Er:YAG laser application causes the shrinkage of mucosa. And there is no production of carbonization, necrosis, or hemorrhage posttherapy. This makes the treatment to be cost effective, fast, and easy with positive results and patient satisfaction.

These findings suggest that the contact Nd:YAG laser might be more effective for soft palate stiffening operations.

## Figures and Tables

**Table 1:** Common symptoms associated with OSA.

Nocturnal	Diurnal
Snoring	Excessive sleepiness
Witnessed Apneas	Morning headaches
Choking at night	Depression/irritability
Nocturia	Memory loss
Insomnia	Decreased libido

**Table 2:** Based on the Apnea-hypopnea index (AHI), and oxygen saturation category for the sleep apnea can be categorized into 3 groups.

	Mild	Moderate	Severe
AHI	5-14	15-30	>30
Oxygen saturation	At least 86%	80% to 85%	<79%

**Table 3:** Study subject demographics.

S.No	Author	Publication year	Study type	Number of subjects	Average age/range	Gender
1.	Usumez et al.	2016	Animal model	20	N/A	Female
2.	Camacho et al.	2017	Systematic review	717	50 ± 9	NA
3.	Lee et al.	2015	Prospective pilot	7	59.5	5 male, 2 female
4.	Camargo et al.	2020	Randomized clinical study protocol trial	36	30-60	NA
5.	Karaman et al.	2017	Prospective nonrandomized clinical study	20	30-56	16 male, 4 female
6.	Yaremchuk	2016	Literature review	NA	NA	NA
7.	Miracki et al.	2013	Randomized clinical trial	57	NA	NA
8.	Neruntarat et al.	2020	Systematic review	247	26-74	NA
9.	Shiffman et al.	2018	Literature review	NA	NA	NA
10.	Sippus	2015	Case report	5	30-56	3 male, 2 female
11.	Storchi et al.	2018	Prospective cohort study	40	53	29 male, 11 female
12.	Wischhusen et al.	2019	Systematic review	3093	NA	NA

**Table 4:** Study based on OSA and snoring with their result measurement variables.

S.No	Author	Publication year	Snoring	OSA	Study variables
1.	Usumez et al.	2016	✓		Histological effect
2.	Camacho et al.	2017		✓	Apnea-hypopnea index (AHI), respiratory disturbance index
3.	Lee et al.	2015	✓	✓	Total airway volume
4.	Camargo et al.	2020		✓	Apnea-hypopnea index
5.	Karaman et al.	2017		✓	Apnea-hypopnea index
6.	Yaremchuk	2016	✓	✓	Apnea-hypopnea index
7.	Miracki et al.	2013	✓		NightLase questionnaire and sleep-disordered breathing score
8.	Neruntarat et al.	2020	✓		Snoring scores, patient satisfaction, AHI, respiratory disturbance index (RDI), Mallampati classification, Epworth sleepiness scale
9.	Shiffman et al.	2018	✓	✓	SnoreLab iPhone application, CBCT
10.	Sippus	2015	✓	✓	Mallampati classification
11.	Storchi et al.	2018	✓		Epworth sleepiness scale, AHI, Mallampati classification
12.	Wischhusen et al.	2019	✓	✓	Complications and side effect

**Table 5:** Type of laser, location, and wavelength.

S.No	Author	Publication year	Laser type	Location	Wavelength
1.	Usumez et al.	2016	Er:YAG laser	Soft palate	2940 nm
2.	Camacho et al.	2017	NA	Uvula	NA
3.	Lee et al.	2015	Nonablative Er:YAG laser	Palatoglossal arch, palatopharyngeal arch, and uvula	2940 nm
4.	Camargo et al.	2020	Diode laser	Soft palate, uvula, pharyngeal walls, palatine tonsils, and on the tongue base	808 nm
5.	Karaman et al.	2017	CO_2_ laser	Tongue base	10,600 nm
6.	Yaremchuk	2016	NA	NA	NA
7.	Miracki et al.	2013	Nonablative Er:YAG	Palate, uvula with the lower part of hard palate, posterior pillars and tonsils, the lateral and bottom sides of the tongue	2940 nm
8.	Neruntarat et al.	2020	Nonablative Er:YAG	Oropharyngeal mucosa, the soft plate, and the tongue	2940 nm
9.	Shiffman et al.	2018	Subablative combined Nd:YAG and Er:YAG	Soft palate and uvula	1064 nm, 2940 nm
10.	Sippus	2015	Er:YAG laser	Soft intraoral tissue	1064 nm and 2940 nm
11.	Storchi et al.	2018	Er.YAG	Soft palate, uvula, tonsillary regions, and base of the tongue	2940 nm
12.	Wischhusen et al.	2019	NA	NA	NA

## References

[1] Eimar H., al-Saleh M. A. Q., Cortes A. R. G., Gozal D., Graf D., Flores-Mir C. (2019). Sleep-Disordered Breathing Is Associated with Reduced Mandibular Cortical Width in Children. *JDR Clinical & Translational Research*.

[2] Park J. G., Ramar K., Olson E. J. (2011). Updates on Definition, Consequences, and Management of Obstructive Sleep Apnea. *Mayo Clinic Proceedings*.

[3] Sleep Apnea (2020). https://www.sleepfoundation.org/sleep-apnea.

[4] Sleep apnea (2018). https://www.mayoclinic.org/diseases-conditions/sleep-apnea/symptoms-causes/syc-20377631.

[5] Slowik J. M., Collen J. F. (2020). Obstructive Sleep Apnea. *StatPearls*.

[6] Bernstein P., Ebba J. H. (2006). Snoring Versus Obstructive Sleep Apnea: A Case Report. *The Permanente Journal*.

[7] Gozal D. (1998). Sleep-disordered breathing and school performance in children. *Pediatrics*.

[8] Jennum P., Ibsen R., Kjellberg J. (2013). Morbidity and mortality in children with obstructive sleep apnoea: a controlled national study. *Thorax*.

[9] Kamami Y. V. (1994). Outpatient treatment of sleep apnea syndrome with CO_2_ Laser, LAUP: Laser-Assisted UPPP results on 46 patients. *Journal of Clinical Laser Medicine & Surgery*.

[10] Littner M., Kushida C. A., Hartse K. (2001). Practice Parameters for the Use of Laser-Assisted Uvulopalatoplasty: An Update for 2000. *Sleep*.

[11] de Camargo F. C. F., DeMoura J. R., Cepeda F. X. (2020). Photobiomodulation by Low-Level Laser Therapy in Patients with Obstructive Sleep Apnea. *Medicine*.

[12] Fini Storchi I., Parker S., Bovis F., Benedicenti S., Amaroli A. (2019). Correction to: Outpatient Erbium: YAG (2940 Nm) Laser Treatment for Snoring: a Prospective Study on 40 Patients. *Lasers in Medical Science*.

[13] Kamami Y. V. (1990). Laser CO2 for snoring. Preliminary results. *Acta Oto-Rhino-Laryngologica Belgica*.

[14] McGuinness L. A., Higgins J. P. T. (2021). Risk-of-bias VISualization (robvis): An R package and Shiny web app for visualizing risk-of-bias assessments. *Research Synthesis Methods*.

[15] Lee C. Y. S., Lee C. C. Y. T. (2015). Evaluation of a Non-Ablative Er: YAG Laser Procedure to Increase the Oropharyngeal Airway Volume: A Pilot Study. *Dental, Oral and Craniofacial Research*.

[16] Wischhusen J., Qureshi U., Camacho M. (2019). Laser-assisted uvulopalatoplasty (LAUP) complications and side effects: A systematic review. *Nature and Science of Sleep*.

[17] Charokopos A., Card M. E., Gunderson C., Steffens C., Bastian L. A. (2018). The Association of Obstructive Sleep Apnea and Pain Outcomes in Adults: A Systematic Review. *Pain Medicine*.

[18] Shiffman H. S., Lukac M. (2018). NightLase®: Minimally Invasive Laser-Assisted Uvulopalatoplasty. *Journal of the Laser and Health Academy*.

[19] Olmos S. R. (2016). Comorbidities of chronic facial pain and obstructive sleep apnea. *Current Opinion in Pulmonary Medicine*.

[20] Hyams K. D., Wignall F. S., Roswell R. (1996). War syndromes and their Evaluation: From the U.S. Civil War to the Persian Gulf War. *Annals of Internal Medicine*.

[21] Higgins D. M., Kerns R. D., Brandt C. A. (2014). Persistent pain and comorbidity among Operation Enduring Freedom/Operation Iraqi Freedom/Operation New Dawn Veterans. *Pain Medicine*.

